# Dissecting the role of soybean rhizosphere-enriched bacterial taxa in modulating nitrogen-cycling functions

**DOI:** 10.1007/s00253-024-13184-5

**Published:** 2024-05-28

**Authors:** Tianshu Wang, Miao Gao, Weiwei Shao, Li Wang, Chunyan Yang, Xing Wang, Shuihong Yao, Bin Zhang

**Affiliations:** 1 State Key Laboratory of Efficient Utilization of Arid and Semi-Arid Arable Land in Northern China, The Institute of Agricultural Resources and Regional Planning, Chinese Academy of Agricultural Sciences, Beijing, 100081 China; 2 The Key Laboratory of Crop Genetics and Breeding of Hebei, Institute of Cereal and Oil Crops, Hebei Academy of Agricultural and Forestry Sciences, Shijiazhuang, 050031 China; 3 Jiangsu Xuhuai Regional Institute of Agricultural Sciences, Xuzhou, 221131 China; 4 College of Resources and Environmental Sciences, Nanjing Agricultural University, Nanjing, 210095 China

**Keywords:** Soybean rhizosphere effect, N cycle, Rhizosphere-enriched taxa, Shared taxa, Site- and variety-specific taxa

## Abstract

**Abstract:**

Crop roots selectively recruit certain microbial taxa that are essential for supporting their growth. Within the recruited microbes, some taxa are consistently enriched in the rhizosphere across various locations and crop genotypes, while others are unique to specific planting sites or genotypes. Whether these differentially enriched taxa are different in community composition and how they interact with nutrient cycling need further investigation. Here, we sampled bulk soil and the rhizosphere soil of five soybean varieties grown in Shijiazhuang and Xuzhou, categorized the rhizosphere-enriched microbes into shared, site-specific, and variety-specific taxa, and analyzed their correlation with the diazotrophic communities and microbial genes involved in nitrogen (N) cycling. The shared taxa were dominated by Actinobacteria and Thaumarchaeota, the site-specific taxa were dominated by Actinobacteria in Shijiazhuang and by Nitrospirae in Xuzhou, while the variety-specific taxa were more evenly distributed in several phyla and contained many rare operational taxonomic units (OTUs). The rhizosphere-enriched taxa correlated with most diazotroph orders negatively but with eight orders including Rhizobiales positively. Each group within the shared, site-specific, and variety-specific taxa negatively correlated with bacterial *amoA* and *narG* in Shijiazhuang and positively correlated with archaeal *amoA* in Xuzhou. These results revealed that the shared, site-specific, and variety-specific taxa are distinct in community compositions but similar in associations with rhizosphere N-cycling functions. They exhibited potential in regulating the soybean roots’ selection for high-efficiency diazotrophs and the ammonia-oxidizing and denitrification processes. This study provides new insights into soybean rhizosphere-enriched microbes and their association with N cycling.

**Key points:**

• *Soybean rhizosphere affected diazotroph community and enriched nifH, amoA, and nosZ.*

• *Shared and site- and variety-specific taxa were dominated by different phyla.*

• *Rhizosphere-enriched taxa were similarly associated with N-cycle functions.*

**Supplementary Information:**

The online version contains supplementary material available at 10.1007/s00253-024-13184-5.

## Introduction

The rhizosphere is the soil surrounding plant roots, which is a microbial hotspot and plays a pivotal role in soil nutrient cycling (Reinhold-Hurek et al. 2015). The rhizosphere microbiota contains a subset of the microbes in bulk soil that are recruited by root exudates and has been well characterized in several crops, including maize, rice, barley, and soybean (Bulgarelli et al. 2015; Edwards et al. 2015; Mendes et al. 2014; Walters et al. 2018). Many beneficial microbes involved in soil nitrogen (N) cycling have been identified in the microbiota enriched in the rhizosphere, including diazotrophs (e.g., *Rhizobium*, *Pseudomonas*), nitrifiers, and denitrifiers (Trivedi et al. 2020). In addition to the microbes that possess beneficial functions, some rhizosphere-enriched taxa also affect nutrient cycling functions through complex interactions within the community (Chepsergon and Moleleki 2023). Understanding the link between rhizosphere-enriched taxa and nutrient cycling function could provide insights into increasing soil health while meeting crop nutrient demands.

Crop roots recruit varied microbial communities under the effects of soil properties and crop genotype, which can be attributed to the differences in native microbial communities in the bulk soil pool and the composition of root exudations, respectively (Kelly et al. 2022; Zhalnina et al. 2018; Zhang et al. 2018). Although the rhizosphere microbial communities may differ in their compositions and functions under these effects, some shared taxa are found across different sites and crop genotypes, which are often called the “core microbiota” (Jiao et al. 2022b; Yeoh et al. 2017). These shared taxa are considered to possess essential functions such as N fixation for crop growth or health maintenance and are less influenced by environmental variability (Castellano-Hinojosa and Strauss 2021; Chang et al. 2022; Xu et al. 2020; Ling et al. 2022). As the rhizosphere effect is under the influence of local soil conditions (Berg and Smalla 2009; Xu et al. 2009), some rhizosphere-enriched taxa are “site-specific,” which are shared across different crop genotypes only in specific locations (Jin et al. 2017). These “site-specific” taxa can be more sensitive to soil and environmental properties and contribute more to crop adaptation to the local environment when compared to the “core microbiota” (Thiergart et al. 2020). Despite the similarly enriched microbial taxa among sites or genotypes, the unique microbial taxa enriched in different varieties are likely linked to the heritable physiological properties of the varieties, such as fungal resistance and N use (Mendes et al. 2018; Zhang et al. 2019). Therefore, within the rhizosphere-enriched microbiota, microbial recruitment may be controlled through different mechanisms and contribute to community function differentially. However, the differences in the taxonomy and function of the rhizosphere-enriched taxa shared across sites and genotypes and those specific to sites or varieties have not been well studied.

Soybean (*Glycine max*) is a major legume crop that can symbiose with rhizobia and fix N from the air (Schultze and Kondorosi 1998). Soybean relies less on synthetic N fertilizer when compared to nonlegume crops and inputs a considerable amount of natural N into the agricultural system, which affects the nutrient cycling function, especially the N-cycling function, of the rhizosphere microbiota (Sánchez and Minamisawa 2019; Tsiknia et al. 2020). In return, the abundance of the taxa involved in the N cycle in the rhizosphere community can also affect the efficiency of N transformation processes in the rhizosphere and thereby influence crop N use efficiency (Ren et al. 2023). Moreover, it was reported that the rhizosphere microbiota showed a close association with rhizobia, for example, the genus *Bacillus* in the soybean rhizosphere was associated with the symbiotic efficiency of rhizobia (Han et al. 2020). Therefore, the N-cycling bacteria of the soybean rhizosphere may be closely associated with rhizosphere-enriched taxa, but information about this association is still limited.

Accordingly, five soybean varieties with different variety types, growth habits, and stem terminations were planted in two fields with different soil types and environmental conditions, and the bacterial and diazotrophic communities and genes involved in the N cycle in rhizosphere and bulk soils were studied. The objectives of this study were (1) to determine the rhizosphere effect on the bacterial community and the microbes involved in the N cycle in different soybean varieties and field conditions; (2) to characterize the rhizosphere-enriched taxa that are shared among sites and varieties (shared taxa), shared among varieties but not sites (site-specific taxa), and unique to each variety (variety-specific taxa) in the soybean rhizosphere; and (3) to explore the potential relationship between the N-cycling function and the shared and site- and variety-specific taxa enriched in the soybean rhizosphere. The results of this study will provide insights into the regulatory mechanisms of the nutrient cycling function in the soybean rhizosphere.

## Materials and methods

### Experimental sites, soybean varieties, and sample collection

The experiment was carried out in Shijiazhuang (37° 56′ N 114° 43′ E) and Xuzhou (33° 37′ N 116° 57′ E) in 2018. The study region has a warm temperate continental monsoon climate. The annual mean temperature and rainfall were 11.6 and 14.0 °C and 500 and 860 mm in Shijiazhuang and Xuzhou, respectively. The soil contains 72.5% sand (0.02–2 mm), 15.5% silt (0.002–0.02 mm), and 12.0% clay (< 0.002 mm) in Shijiazhuang and 49.7% sand, 30.6% silt, and 19.7% clay in Xuzhou. For both sites, wheat (*Triticum aestivum* L.) (sown in October and harvested in June) was rotated with soybean (sown in June and harvested in October) in a double-cropping system for more than 5 years before the experiment.

The field experiment was laid out following a randomized complete block design with five soybean varieties as treatments and four replicated for each treatment at both experimental sites. Each plot was 1.6 m wide and 5.5 m long. The five soybean varieties selected had different types, growth habits, and stem terminations according to the SoyFGB v2.0 platform (Supplemental Table S1) (Zheng et al. 2022). Soybean seeds were sown manually by 0.4 * 0.11 m^2^ spacing on 16 and 12 June in Shijiazhuang and Xuzhou, respectively. The fertilizer was applied after sowing at the rate of 22.5 kg N ha^−1^ (applied as urea, 46% N), 34.5 kg P_2_O_5_ ha^−1^ (applied as calcium superphosphate, 12% P_2_O_5_), and 34.5 kg K_2_O ha^−1^ (applied as potassium sulfate, 50% K_2_O) for each site and each plot. Pesticides were applied the same as local cropping management. No irrigation was applied and weeds were controlled manually. The soybeans were not inoculated with rhizobia prior or during planting.

Five bulk soil samples were collected between soybean plant rows at approximately 20 cm away from soybean plants and 0–20 cm depth following the five-point sampling method at each site. One plant from each plot (four plants for each variety at each site) except the side row was randomly selected and excavated with roots and the surrounding soil at each site at the full bloom stage. The plants were shaken vigorously to remove the loosely adhered soil on the roots, and the roots together with rhizosphere soil were collected into an ethylene-oxide-sterilized ziplock bag (BKMAM, Changde, China). All samples were immediately transferred to the laboratory with ice packs. The rhizosphere soil was manually separated from the roots using a small brush according to Zhang et al. (2017). After removing fine residues and stones, each soil sample was divided into three parts. One part was stored at − 20 °C for DNA extraction; one was stored at 4 °C for the determination of pH and the contents of water, ammonia, and nitrate; and the remaining soil was air-dried, ground, and sieved to determine the contents of soil organic carbon, total nitrogen, and available phosphorous and potassium.

### Measurement of soil properties

Soil pH was measured using a pH meter (PB-10, Sartorius, Göttingen, Germany) at a soil to distilled water ratio of 1:5 (weight/volume). The soil water content was measured by oven drying at 105 °C until a constant weight was achieved. Soil organic carbon and total N contents were measured using an elemental analyzer (Vario MACRO Cube, Elementar, Hanau, Germany). Soil nitrate and ammonia were extracted using 1 M KCl, and their contents were measured using a continuous flow autoanalyzer (Seal Auto Analyser 3, Seal Analytical, Norderstedt, Germany). The available phosphorous was extracted using 1 M NaHCO_3_, and its content was measured following the molybdenum-blue colorimetry method. The available potassium was extracted using 1 M NH_4_Ac, and its content was measured using a flame photometer (FP640, INASA, Shanghai, China).

### Soil DNA extraction and amplicon sequencing

Total soil DNA was extracted from 0.5 g of each bulk and rhizosphere soil sample using the Fast DNA SPIN kit (MP Biomedicals, Irvine, CA, USA) following the manufacturer’s instructions. The integrity and concentration of the extracted DNA samples were evaluated using 1% agarose gels. The V4 hypervariable region of the 16S rRNA gene and the *nifH* gene fragment were amplified using general universal primer sets 515F/806R (5′-GTGYCAGCMGCCGCGGTAA-3′/5′-GGACTACNVGGGTWTCTAAT-3′) and polF/polR (5′-TGCGAYCCSAARGCBGACTC-3′/5′-ATSGCCATCATYTCRCCGGA-3′), respectively, with Phusion high-fidelity DNA polymerase (New England Biolabs, Ipswich, MA, USA). The polymerase chain reaction (PCR) conditions consisted of an initial denaturation at 98 °C for 1 min, 30 cycles of denaturation at 98 °C for 10 s, annealing at 50 °C for 30 s, elongation at 72 °C for 30 s, and a final extension at 72 °C for 5 min. The PCR products were assessed on a 2% agarose gel, pooled in equidensity ratios, and purified with a GeneJET Gel Extraction kit (Thermo Scientific, Waltham. MA, USA). The sequencing libraries were constructed using a TruSeq DNA PCR-Free Library Preparation kit (Illumina, San Diego, CA, USA). The quality of the libraries was assessed on the Qubit 2.0 Fluorometer (Thermo Scientific, Waltham. MA, USA) and Agilent Bioanalyzer 2100 system (Agilent Technologies, Santa Clara, CA, USA). The libraries were sequenced on an Illumina NovaSeq platform, and 250-bp paired-end reads were generated. The quality of the paired-end reads was assessed with FastQC v.0.11.5 (Andrews 2014). The raw sequences were deposited in the NCBI repository under BioProject accession number PRJNA1045824.

By using USEARCH v.11.0 (Edgar 2010), the paired-end reads were merged (-fastq_mergepairs), the barcode and primer sequences were trimmed (-fastx_truncate), low-quality reads were filtered (-fastq_filter), and the unique sequences were determined (-fastx_uniques) and clustered into operational taxonomic units (OTUs) with ≥ 97% similarity (-cluster_otus). For the V4 region of the 16S rRNA gene, the representative sequence of each OTU was aligned to the SILVA 123 database (Quast et al. 2012) for taxonomic annotation, and FAPROTAX v.1.2.4 (Louca et al. 2016) was used for functional annotation. For the *nifH* gene fragment, sequences were analyzed online at http://cloud.magigene.com/ and aligned with the *nifH* gene database (http://fungene.cme.msu.edu/FunGene) for taxonomic annotation.

### Quantitative PCR (qPCR) of N-cycling genes

The abundances of seven genes involved in N cycling were quantified by qPCR in this study. The name and function of the genes and the primer sets used are shown in Supplemental Table S2. The fragments of each gene were amplified from a random total soil DNA sample, ligated to the pGEM-T vector (Promega, Madison, WI, USA), and then transformed into *Escherichia coli* strain DH5α (Tiangen, Beijing, China). The cloning vector of each gene was screened by culturing on LB medium with IPTG (isopropyl ß-D-1-thiogalactopyranoside) and X-gal according to the method described by Green and Sambrook (2019) and confirmed by PCR amplification with the corresponding primer sets and sequencing analysis. The concentration of the extracted cloning vector of each gene was quantified using a NanoDrop spectrophotometer (NanoDrop, PeqLab Biotechnologie, Erlangen, Germany) and then calculated using the following equation:

1 $$\mathrm{Copies}\;\mathrm{of}\;\mathrm{the}\;\mathrm{cloning}\;\mathrm{vector}\;\mathrm{per}\;\mu L=\mathrm{ng}\;\mathrm{cloning}\;\mathrm{vector}\;\mathrm{per}\;\mu L\times6.02\times10^{14}/N\times660$$

where *N* is the number of base pairs contained in the cloning vector. The quantified cloning vector of each gene was diluted to a tenfold dilution series from 10 to 10^7^ copies μL^−1^, which were later used as standards. The total soil DNA samples were quantified and then diluted to a concentration of approximately 100 ng µL^−1^ before being used as qPCR templates.

qPCR was performed on an Applied Biosystems 7500 Real-Time System (Life Technologies, Carlsbad, CA, USA) in a 20-μL mixture containing 10 μL of TB Green *Premix Ex Taq* II (Tli RNaseH Plus) (TaKaRa, Otsu, Japan), 0.4 μM of each forward and reverse primer for each gene, 0.4 μL of ROX reference dye II (50 ×), 2 μL of DNA template, and 6 μL of ddH_2_O. The qPCR conditions consisted of an initial denaturation at 95 °C for 30 s, 40 cycles of 95 °C for 5 s and 60 °C for 34 s, and a dissociation stage. All of the total soil DNA samples and standards were run in triplicate for technical replicates. Standard curves were generated by regressing the standard’s copy number with its cycle threshold (Ct) value. The copy number of each gene in each soil sample was determined by the mean Ct value of the three technical replicates using the corresponding standard curves. The absolute abundance of a gene refers to the copy number per gram soil dry weight.

### Statistical analysis

The statistical analyses were performed using R software (version 4.0.2, https://www.r-project.org/). Two-way ANOVA followed by Tukey’s HSD test was performed to determine the effects of soybean rhizosphere and variety on soil properties, the alpha diversity indices, and the relative abundances of the top 10 most abundant phyla and top 20 most abundant orders of bacterial communities. For alpha diversity indices of bacterial communities, richness was calculated as the number of OTUs observed in each sample, the Shannon index was calculated using the *diversity* function in the *vegan* package (https://cran.r-project.org/web/packages/vegan/index.html), and the phylogenetic diversity (PD) index of the whole tree was calculated using the *pd* function in the *picante* package (https://cran.r-project.org/web/packages/picante/index.html). For the beta diversity of bacterial and diazotrophic communities, Bray‒Curtis dissimilarity among samples was calculated using the *vegdist* function in the *vegan* package (https://cran.r-project.org/web/packages/vegan/index.html), and the effects of the experimental site, compartment, and soybean variety were visualized by principal coordinate analysis (PCoA) and constrained PCoA (CPCoA). A permutational multivariate ANOVA (PERMANOVA) with 999 random permutations was performed using the *Adonis* function in *vegan* to determine the significance of the effects. The differences between the relative abundance of each OTU in the bulk and rhizosphere soil bacterial communities were assessed using the *DESeq2* package (https://bioconductor.org/packages/release/bioc/html/DESeq2.html) at FDR (false discovery rate)-adjusted *p* < 0.05 and estimated log2-fold change > 1. The Venn diagrams of the enriched OTUs were generated online at https://bioinformatics.psb.ugent.be/webtools/Venn/ to identify the shared, site-specific, and variety-specific taxa. The phylogenetic trees were generated using the online tool iTOL (https://itol.embl.de/) (Letunic and Bork 2021). The co-occurrence networks between the diazotrophic orders and the OTUs in the bacterial community were constructed using the *ggClusterNet* package (Wen et al. 2022) with *r* and *p* thresholds of 0.6 and 0.05, respectively. Student’s *t* test was performed to test the difference between the absolute abundance of N-cycling genes in bulk and rhizosphere soils at each site at *p* < 0.05. The random forest model was applied to assess the importance and significance of the effects of each OTU on the community N-cycling function using the *rfPermute* package (https://cran.r-project.org/web/packages/rfPermute/index.html). Spearman correlation and Mantel tests were performed to analyze the correlation of the shared, site-specific, and variety-specific taxa with soil properties and the abundance of N-cycle-related genes using the *correlate* and *Mantel_test* functions in the *linkET* package (https://github.com/Hy4m/linkET), respectively.

## Results

### Soil properties

Soil properties were different between Shijiazhuang and Xuzhou, bulk and rhizosphere soil, and among soybean varieties (Table 1). The soil water content, organic carbon, and total N were significantly (*p* < 0.05) lower, while ammonium and available potassium were significantly higher in both bulk and rhizosphere soil in Shijiazhuang than in Xuzhou. The soil pH and organic carbon, total N, nitrate, and available potassium contents were significantly higher, and the water content was significantly lower in the soybean rhizosphere than in the bulk soil at both sites. The soil ammonium content was significantly lower in the rhizosphere than in the bulk soil in Xuzhou, and the available phosphorus content in the rhizosphere was significantly higher in Shijiazhuang but significantly lower in Xuzhou than in the bulk soil. Although all soil properties except for soil pH were significantly different among the rhizosphere soils of the five soybean varieties in at least one site, none of them exhibited a consistent pattern of variation between the two sites.

**Table 1:** Selected soil properties in the bulk soil and the rhizosphere of the five soybean varieties at Shijiazhuang and Xuzhou

Site	Compartment	Variety	Soil properties^a^							
			SWC	pH	SOC	STN	NO_3_^−^	NH_4_^+^	AP	AK
			%		g kg^−1^		mg kg^−1^			
Shijiazhuang station (37° 56′ N 114° 43′ E)										
Bulk soil			12.4 ± 0.6A	7.9 ± 0.1B	16.8 ± 1.2B	1.05 ± 0.06B	30.8 ± 8.5A	3.12 ± 0.21AB	16.9 ± 0.3C	95.7 ± 5.1C
Rhizosphere soil										
		DF	12.0 ± 0.2A	8.3 ± 0.0A	22.6 ± 1.9A	1.36 ± 0.10A	24.2 ± 2.0A	3.63 ± 0.43AB	65.7 ± 10.7A	113.3 ± 7.3BC
		HJ	11.2 ± 0.3A	8.2 ± 0.0AB	19.1 ± 0.4AB	1.06 ± 0.06B	48.7 ± 10.6A	5.42 ± 0.23A	53.1 ± 6.2AB	138.61 ± 11.4AB
		MC	12.6 ± 0.6A	8.1 ± 0.1AB	21.9 ± 0.8A	1.19 ± 0.01AB	37.4 ± 11.4A	1.98 ± 0.11B	53.2 ± 3.9AB	162.8 ± 7.8A
		CX	10.5 ± 0.7A	8.3 ± 0.1A	19.2 ± 0.7AB	1.21 ± 0.07AB	46.9 ± 8.7A	4.61 ± 1.09AB	37.0 ± 3.7BC	119.3 ± 8.7BC
		QX	12.8 ± 0.7A	8.4 ± 0.1A	21.6 ± 0.8A	1.27 ± 0.03AB	32.3 ± 5.6A	5.42 ± 0.90A	25.3 ± 2.6C	126.0 ± 4.2BC
Xuzhou (33° 37′ N 116° 57′ E)										
Bulk soil			16.8 ± 0.7ab	7.9 ± 0.1b	22.5 ± 1.0c	1.27 ± 0.13b	13.1 ± 1.6b	3.35 ± 0.27a	33.0 ± 4.4a	119.4 ± 12.5a
Rhizosphere soil										
		DF	18.0 ± 0.8a	8.3 ± 0.0a	21.7 ± 0.3c	1.23 ± 0.04b	33.8 ± 6.6ab	2.57 ± 0.10b	22.2 ± 1.7b	128.8 ± 8.2a
		HJ	14.9 ± 0.7abc	8.2 ± 0.0a	27.8 ± 0.2a	1.74 ± 0.03a	57.2 ± 12.2a	2.23 ± 0.11b	29.9 ± 2.1ab	116.8 ± 24.2a
		MC	12.6 ± 0.8c	8.3 ± 0.0a	25.1 ± 0.2b	1.70 ± 0.06a	36.7 ± 3.3ab	2.30 ± 0.15b	26.5 ± 1.1ab	110.4 ± 7.0a
		CX	14.6 ± 1.3abc	8.1 ± 0.1a	26.4 ± 0.3ab	1.76 ± 0.09a	25.3 ± 0.7b	2.24 ± 0.10b	24.9 ± 1.7ab	178.9 ± 31.0a
		QX	13.7 ± 0.3bc	8.2 ± 0.0a	26.3 ± 0.6ab	1.80 ± 0.07a	22.2 ± 2.3b	2.07 ± 0.08b	29.5 ± 1.9ab	170.3 ± 33.2a
Two-way ANOVA (*p* value): effects of experiment site and compartment										
Site			** < 0.001**	0.396	** < 0.001**	** < 0.001**	0.221	** < 0.001**	** < 0.001**	0.256
Compartment			**0.046**	** < 0.001**	** < 0.001**	** < 0.001**	**0.017**	0.953	0.009	**0.031**
Site × compartment			0.263	0.555	0.522	0.151	0.211	**0.016**	** < 0.001**	0.576

^a^*SWC* soil water content, *SOC* soil organic carbon, *STN* soil total nitrogen, *AP* available phosphorus, *AK* available potassium. ± refers to standard error. Different letters indicate significant differences among the varieties at each site at *p* < 0.05 according to Tukey’s HSD test

The values in bold indicate signigicant effects of experiment site and compartment at *p* < 0.05 according to Two-way ANOVA

### Bacterial communities

**Fig. 1:**
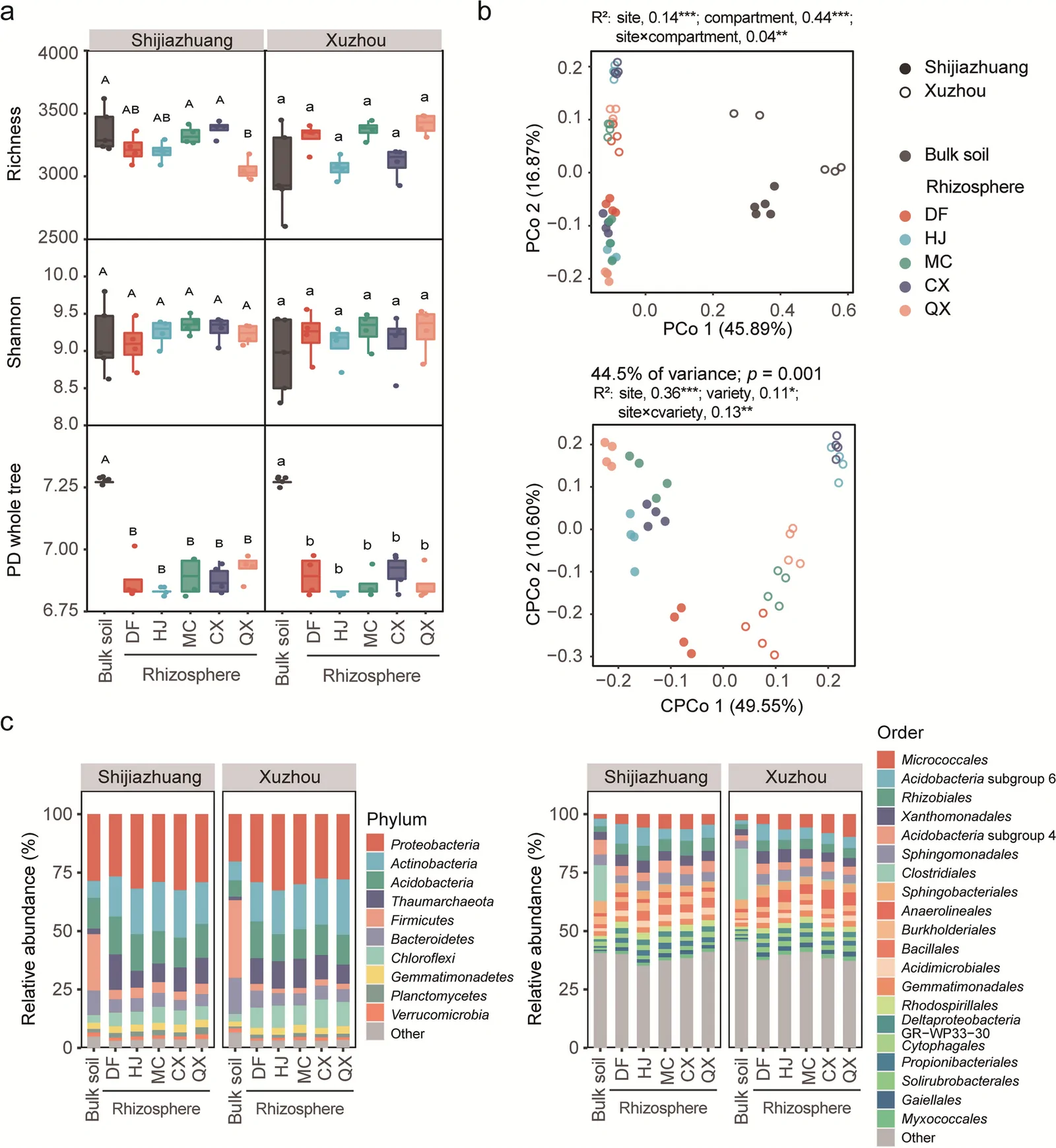
Bacterial communities in bulk soil and the rhizosphere of the five soybean varieties at Shijiazhuang and Xuzhou. **a** The richness, Shannon, and PD whole tree indices. Different letters indicate significant differences at *p* < 0.05; **b** unconstrained PCoA of the whole bacterial data and constrained PCoA of the data of rhizosphere soil in Bray‒Curtis distance. *, **, and *** indicate *p* < 0.05, 0.01, and 0.001, respectively; **c** the relative abundance of the top 10 most abundant phyla and top 20 most abundant orders

The soybean rhizosphere effect strongly influenced the diversity and composition of soil bacterial communities in Shijiazhuang and Xuzhou (Fig. 1). The PD whole tree index of the rhizosphere soil for the five cultivars was significantly (*p* < 0.05) lower than that in the bulk soil at both sites, while the richness and Shannon indices in rhizosphere soil except for QX were similar to those in bulk soils (Fig. 1a). The richness in the rhizosphere of QX was significantly lower than that in bulk soils in Shijiazhuang. The PCoA and CPCoA based on Bray‒Curtis dissimilarity showed significant effects of the experimental site, sample compartment, and soybean variety on the bacterial communities (Fig. 1b). When both bulk and rhizosphere soil samples were included, the sample compartment explained 44% of the variance (*p* < 0.001), followed by the experimental site (*R*^2^ = 0.14, *p* < 0.001), and their interactive effect was very small (*R*^2^ = 0.04, *p* < 0.01). CPCoA was further performed on the rhizosphere samples, which showed small but significant effects of soybean variety (*R*^2^ = 0.11, *p* < 0.05) and its interaction with the experimental site (*R*^2^ = 0.13, *p* < 0.01) on the rhizosphere bacterial communities.

The bacterial community composition was significantly affected by the experimental site, compartment, and soybean variety (Fig. 1c, Supplemental Table S3). At the phylum level, *Proteobacteria* (24.4%) and *Firmicutes* (28.7%) were the most dominant in bulk soil, and *Proteobacteria* (29.7%) and *Actinobacteria* (19.5%) were the most dominant in rhizosphere soil at both sites. Among the top 10 most abundant phyla, *Proteobacteria*, *Actinobacteria*, *Acidobacteria*, *Thaumarchaeota*, *Chloroflexi*, *Gemmatimonadetes*, and *Planctomycetes* were significantly more abundant in rhizosphere soil than in bulk soil, and *Firmicutes*, *Bacteroidetes*, and *Verrucomicrobia* were significantly more abundant in bulk soil than in rhizosphere soil. *Bacteroidetes*, *Chloroflexi*, and *Planctomycetes* were significantly affected by site, and *Chloroflexi* in the rhizosphere soil was significantly affected by variety. Among the top 20 most abundant orders, nine orders, including *Rhizobiales*, *Bacillales*, *Acidimicrobiales*, *Rhodospirillales*, *Deltaproteobacteria* GR-WP33-30, *Propionibacteriales*, *Solirubrobacterales*, *Gaiellales*, *Myxococcales*, and *Micrococcales*, were significantly enriched in the soybean rhizosphere for all the five soybean varieties at both sites (Supplemental Table S4).

### Shared and site- and variety-specific taxa enriched in the soybean rhizosphere

**Fig. 2:**
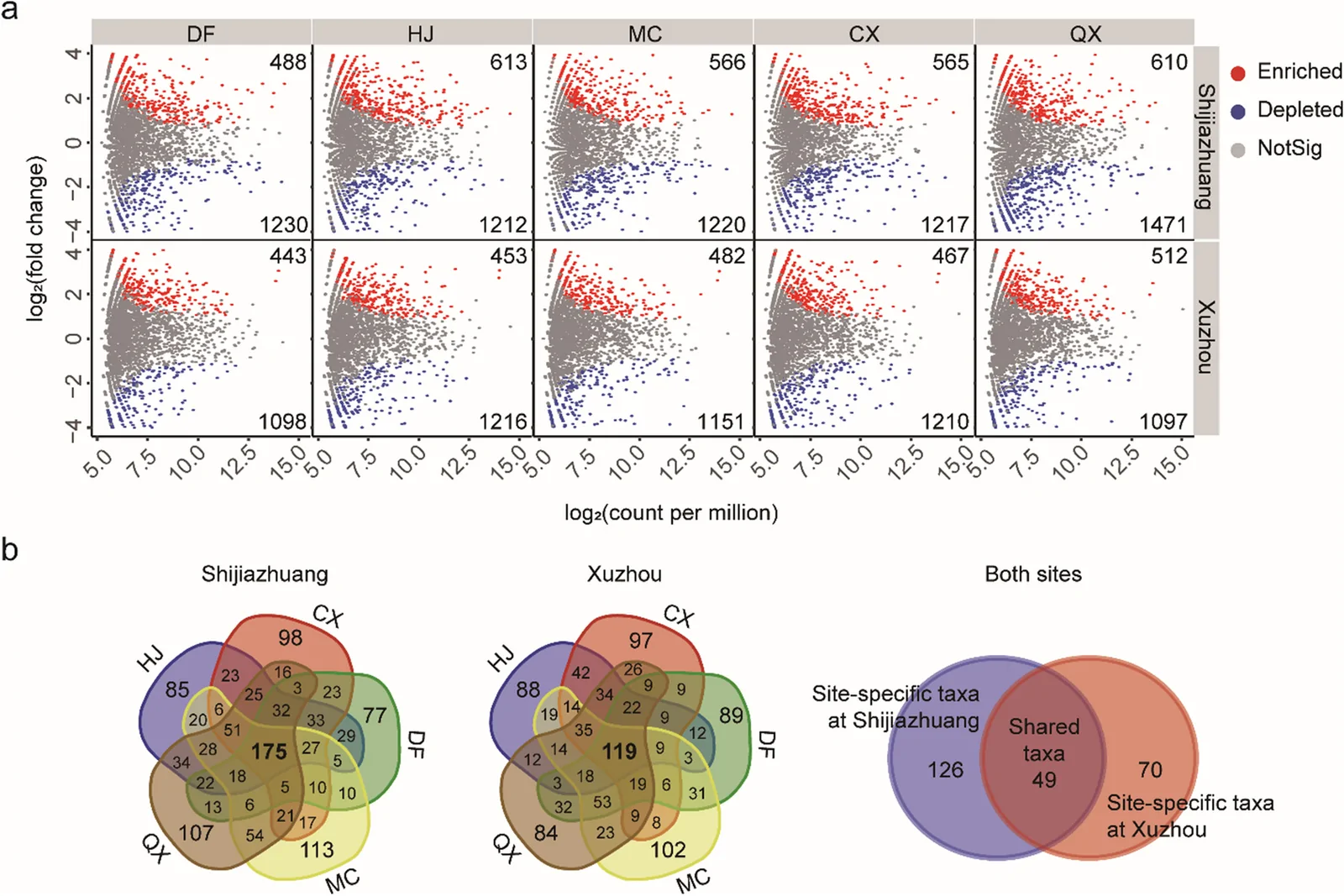
OTUs significantly (adjusted *p* < 0.1) enriched or depleted in the rhizosphere of each soybean variety compared to the bulk soil at each site (**a**) and Venn diagrams of the enriched OTUs (**b**)

The rhizosphere-enriched taxa were identified by comparing the bacterial community present in the rhizosphere of each soybean variety with that in the bulk soil at each site. Based on their intersections, these OTUs were then classified into three categories: shared, site-specific, and variety-specific taxa (Fig. 2). The number of OTUs enriched in the soybean rhizosphere ranged from 443 to 613, which was lower than the number of depleted OTUs (Fig. 2a). The 49 enriched OTUs that were shared across all sites and varieties were classified as shared taxa; the 126 and 70 enriched OTUs that were shared among soybean varieties but not sites were classified as site-specific taxa in Shijiazhuang and Xuzhou, respectively; and the rhizosphere-enriched OTUs that were unique to each soybean variety were classified as variety-specific taxa (Fig. 2b).

**Fig. 3:**
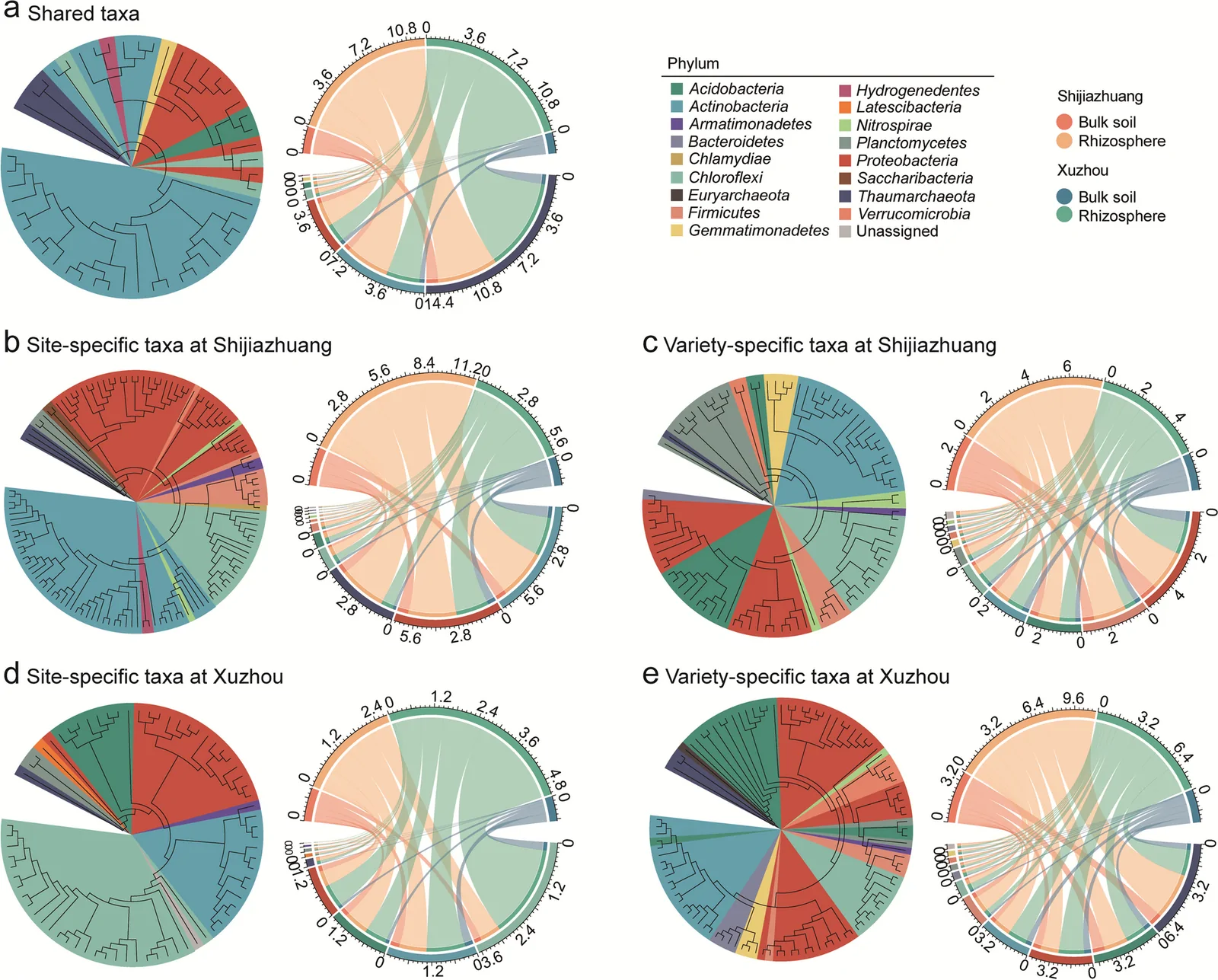
Phylogenetic relationships and relative abundances of rhizosphere-enriched taxa shared across varieties and sites (**a**), site-specific taxa at **b** Shijiazhuang and **d** Xuzhou, and variety-specific taxa at **c** Shijiazhuang and **e** Xuzhou

The shared, site-specific, and variety-specific taxa showed differences in the composition of both OTU taxonomy and relative abundance, although they were similarly dominated by *Actinobacteria* and *Proteobacteria* (Fig. 3). Among the shared taxa, most of the OTUs belonged to *Actinobacteria* (63.3%) and *Proteobacteria* (16.3%), and *Thaumarchaeota* and *Actinobacteria* were the phyla with the highest relative abundances (Fig. 3a). In Shijiazhuang, *Nitrospirae* was the third most dominant phylum by OTU numbers in both site-specific and variety-specific taxa, but it was relatively low in abundance (Fig. 3b, c). *Thaumarchaeta* was the third most abundant phylum after *Actinobacteria* and *Proteobacteria* among site-specific taxa, while *Proteobacteria* and *Firmicutes* were the most abundant among variety-specific taxa. In Xuzhou, *Nitrospirae* was the most dominant phylum by both OTU number and relative abundance among site-specific taxa (Fig. 3d). *Thaumarchaeota* and *Acidobacteria* were the most abundant phyla with higher relative abundances than *Actinobacteria* and *Proteobacteria* among variety-specific taxa (Fig. 3e). Notably, the variety-specific taxa were widely distributed in 21 phyla in both Shijiazhuang and Xuzhou, and 391 out of 480 OTUs in Shijiazhuang and 358 out of 460 OTUs in Xuzhou were rare taxa with relative abundances less than 0.1‰ (Supplemental Table S5).

### Diazotroph community and its correlation with shared and site- and variety-specific taxa enriched in the soybean rhizosphere

**Fig. 4:**
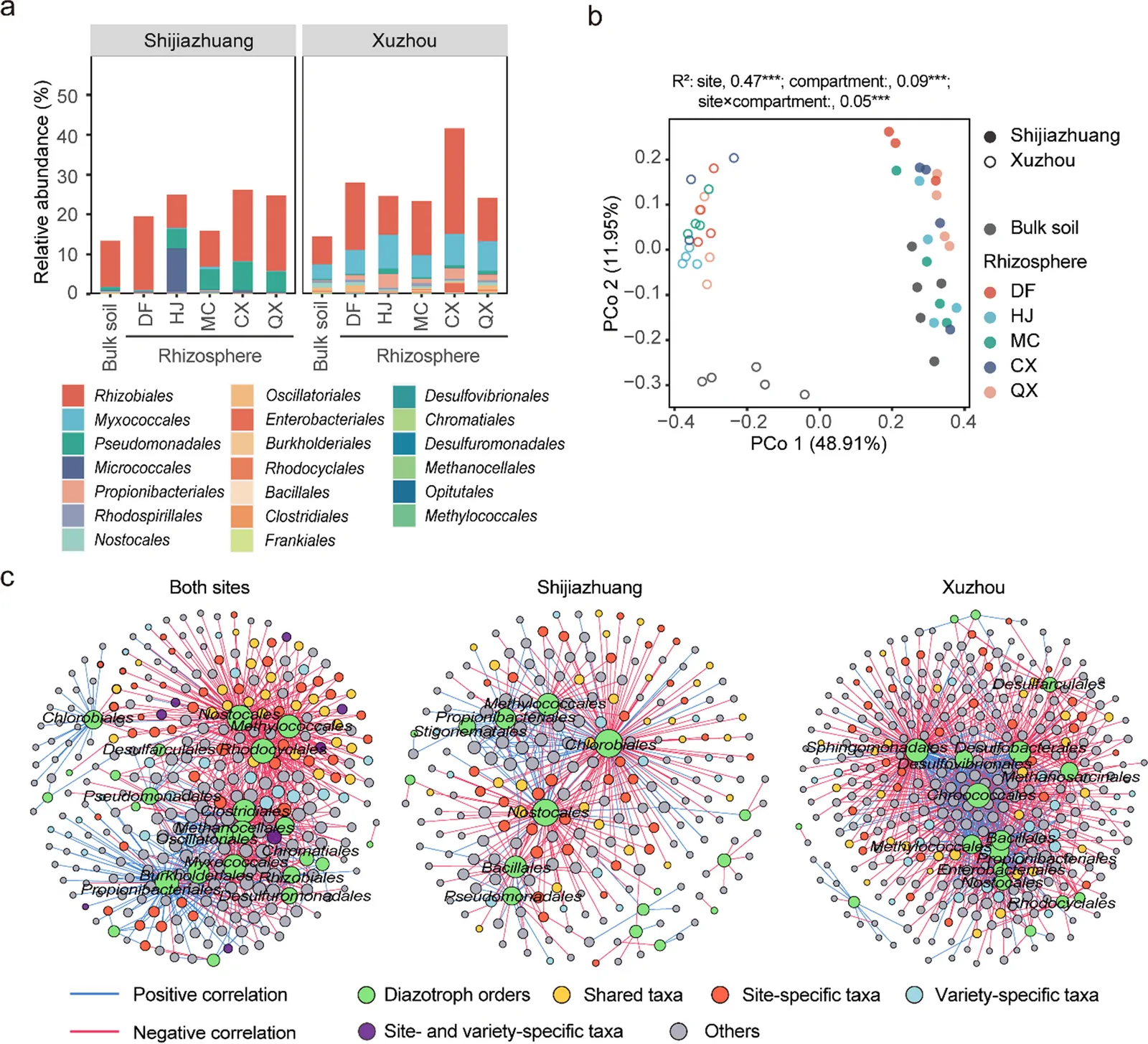
The diazotrophic community and its correlation with shared and site- and variety-specific taxa enriched in the soybean rhizosphere. **a** The relative abundance of the top 20 orders in the diazotroph community in bulk soil and the rhizosphere of five cultivars in Shijiazhuang and Xuzhou; **b** PCoA of the diazotroph community in Bray‒Curtis distance; **c** the co-occurrence network between diazotroph orders and site- and variety-specific taxa enriched in the soybean rhizosphere. Orders with a degree higher than 10 were labeled

The diazotroph community composition was different between rhizosphere and bulk soil and between Shijiazhuang and Xuzhou (Fig. 4a). At the order level, *Rhizobiales* was the most abundant at both sites. *Myococcales* was more abundant in Xuzhou, while *Pseudomonadales* and *Micrococcales* were more abundant in Shijiazhuang. At Shijiazhuang, *Pseudomonadales* was enriched in the rhizospheres of four out of five varieties (HJ, MC, CX, and QX), and *Micrococcales* was enriched only in the rhizosphere of HJ. At Xuzhou, the top five most abundant orders, including *Rhizobiales*, *Myococcales*, *Pseudomonadales*, *Micrococcales*, and *Propionibacteriales*, were more abundant in the soybean rhizosphere than in the bulk soil. Unlike the total bacterial community, the diazotrophic community was mainly affected by the experimental site (PERMANOVA: *R*^2^ = 0.47, *p* < 0.001), and the effect of compartment (PERMANOVA: *R*^2^ = 0.08, *p* < 0.001) was smaller than that of site (Fig. 4b). The diazotrophic community was separated by experimental site at PCo 1, while the communities in bulk soil and rhizosphere soil were separated at PCo 2 only in Xuzhou.

Co-occurrence interaction networks were constructed between the diazotroph orders and the OTUs in bacterial communities (Fig. 4c). In the both-site network, the shared and site- and variety-specific taxa contributed 294 out of the 888 total edges. The average degrees of the shared and site- and variety-specific taxa were 2.84, 2.90, and 3.40, respectively. More negative correlations (652 edges) than positive ones (236 edges) were found in the both-site network, but more positive than negative correlations were found for *Propionibacterials*, *Burkholderiales*, *Chlorobiales*, and *Rhizobiales*. In the Shijiazhuang network, a total of 502 edges (263 negative and 139 positive) were found, and shared, site-specific, and variety-specific taxa were linked with diazotrophic orders through 41, 70, and 19 edges, respectively. They were negatively correlated with *Chlorobiales*, *Nostocales*, *Methylococcales*, and *Bacillales* and positively correlated with *Propionibacteriales* and *Pseudomonadales*. In the Xuzhou network, a total of 969 edges (645 negative and 324 positive) were found, and shared, site-specific, and variety-specific taxa were linked with diazotrophic orders through 70, 91, and 79 edges, respectively. They were positively correlated with *Propionibacteriales*, *Enterobacteriales*, and *Rhizobiales* and negatively correlated with the remaining diazotrophic orders.

### The N-cycling function and its relationship with shared and site- and variety-specific taxa enriched in the soybean rhizosphere

**Fig. 5:**
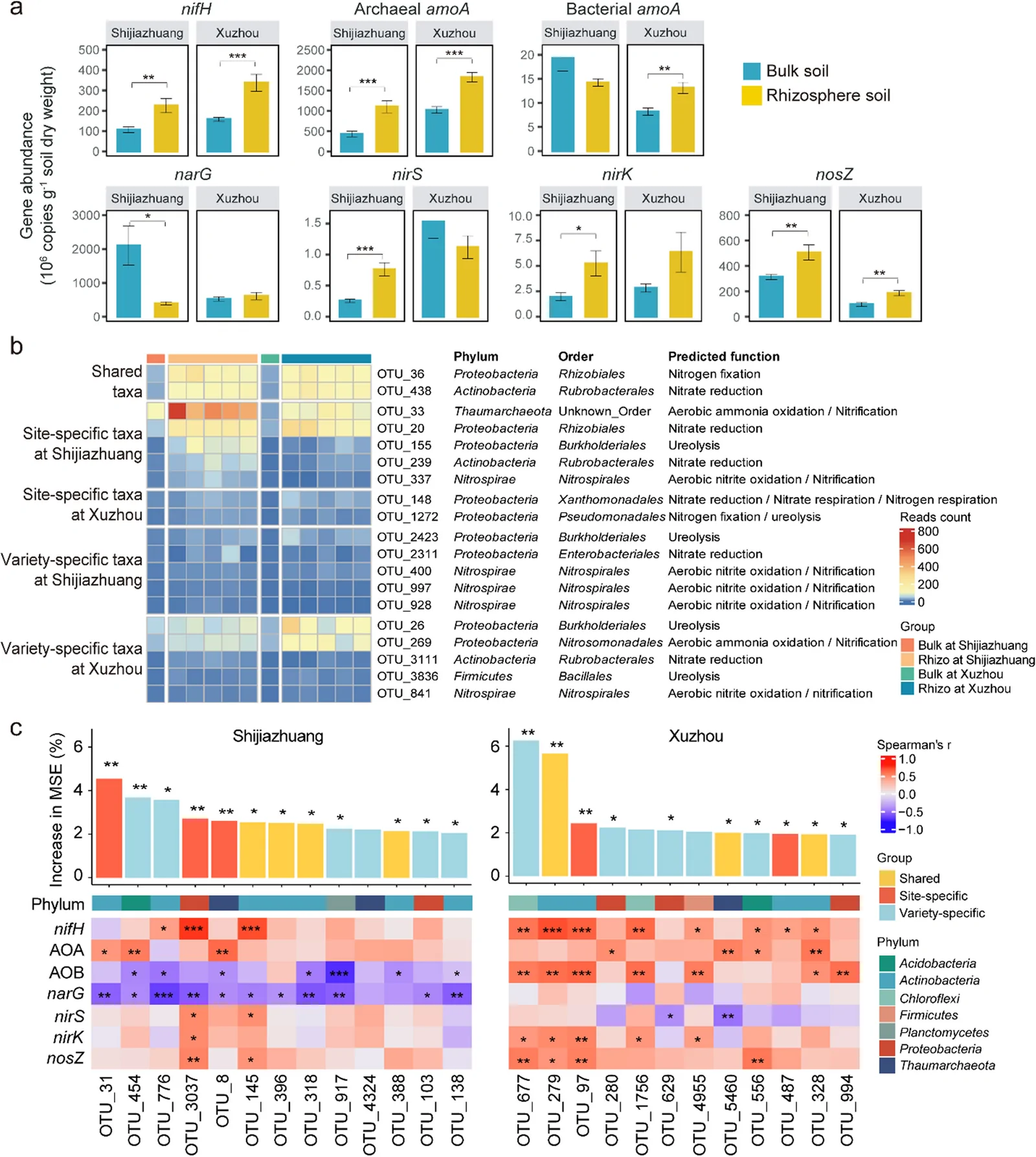
The abundance of N-cycle-related genes and the relationships between the rhizosphere-enriched taxa and N-cycle-related genes. **a** The absolute abundance of N-cycle-related genes in the bulk soil and soybean rhizosphere. Error bars represent standard error; **b** the read count of the rhizosphere-enriched OTUs with predicted N-cycle functions; **c** random forest models of the rhizosphere-enriched OTUs predicting N-cycle function and Spearman’s correlation between the OTUs and the abundance of N-cycle-related genes. The OTUs with the top 13 and 12 increases in mean squared error (MSE) were visualized at Shijiazhuang and Xuzhou, respectively, as determined by the tenfold cross-validation error (Supplemental Fig. S2). *, **, and ** indicate significant differences at *p* < 0.05, 0.01, and 0.001, respectively

Seven genes involved in N cycling were quantified to determine their absolute abundance in bulk soil and soybean rhizosphere soil, and six of them were enriched in the soybean rhizosphere (Fig. 5a). The abundances of *nifH* (involved in N fixation), archaeal *amoA* (involved in ammonia oxidation), and *nosZ* (involved in N_2_O reduction) were significantly (*p* < 0.05) higher in the soybean rhizosphere than in the bulk soil at both sites. The abundances of bacterial *amoA* in Xuzhou and nitrite reductase genes *nirS* and *nirK* in Shijiazhuang were significantly higher in the soybean rhizosphere than in the bulk soil. The abundance of *narG* (involved in nitrate reduction) was significantly lower in the soybean rhizosphere than in the bulk soil in Shijiazhuang. A differentiation between bulk and soybean rhizosphere soil was also observed in the predicted N-cycling functions (Supplemental Fig. S1). The predicted N-cycling functions were generally enriched in the soybean rhizosphere at both sites, except for nitrate denitrification and nitrite denitrification and respiration in Shijiazhuang and nitrate reduction at both sites.

To determine the contribution of the shared, site-specific, and variety-specific taxa to the community N-cycling functions, the function of each OTU was predicted, and their correlation with the abundance of N-cycle-related genes was examined (Fig. 5b, c). Only 19 OTUs were annotated with N-cycle-related functions, and they belong to the phyla *Proteobacteria*, *Actinobacteria*, *Thaumarchaeota*, *Nitrospirae*, and *Firmicutes* (Fig. 5b). Among the 19 OTUs, 11 OTUs had read counts larger than 50 (relative abundance > 1‰), and they possessed the functions of ureolysis, nitrate reduction, ammonia oxidation, nitrite oxidation, nitrification, and N fixation. According to the random forest analyses and the cross-validation curves, the 13 and 12 most important OTUs at Shijiazhuang and Xuzhou were correlated with the abundance of each N-cycle-related gene, respectively (Fig. 5c). In Shijiazhuang, four shared, three site-specific, and six variety-specific OTUs were included. Most of the OTUs were negatively correlated with bacterial *amoA* and *narG*, and less than half of the OTUs were positively correlated with *nifH*, archaeal *amoA*, *nirS*, *nirK*, and *nosZ*. In Xuzhou, three shared, two site-specific, and seven variety-specific OTUs were included. More significant (*p* < 0.05) positive correlations were found than in Shijiazhuang, where more than half of the OTUs were positively correlated with *nifH* and bacterial *amoA*, and negative correlations were found only between *nirS* and two OTUs.

**Fig. 6:**
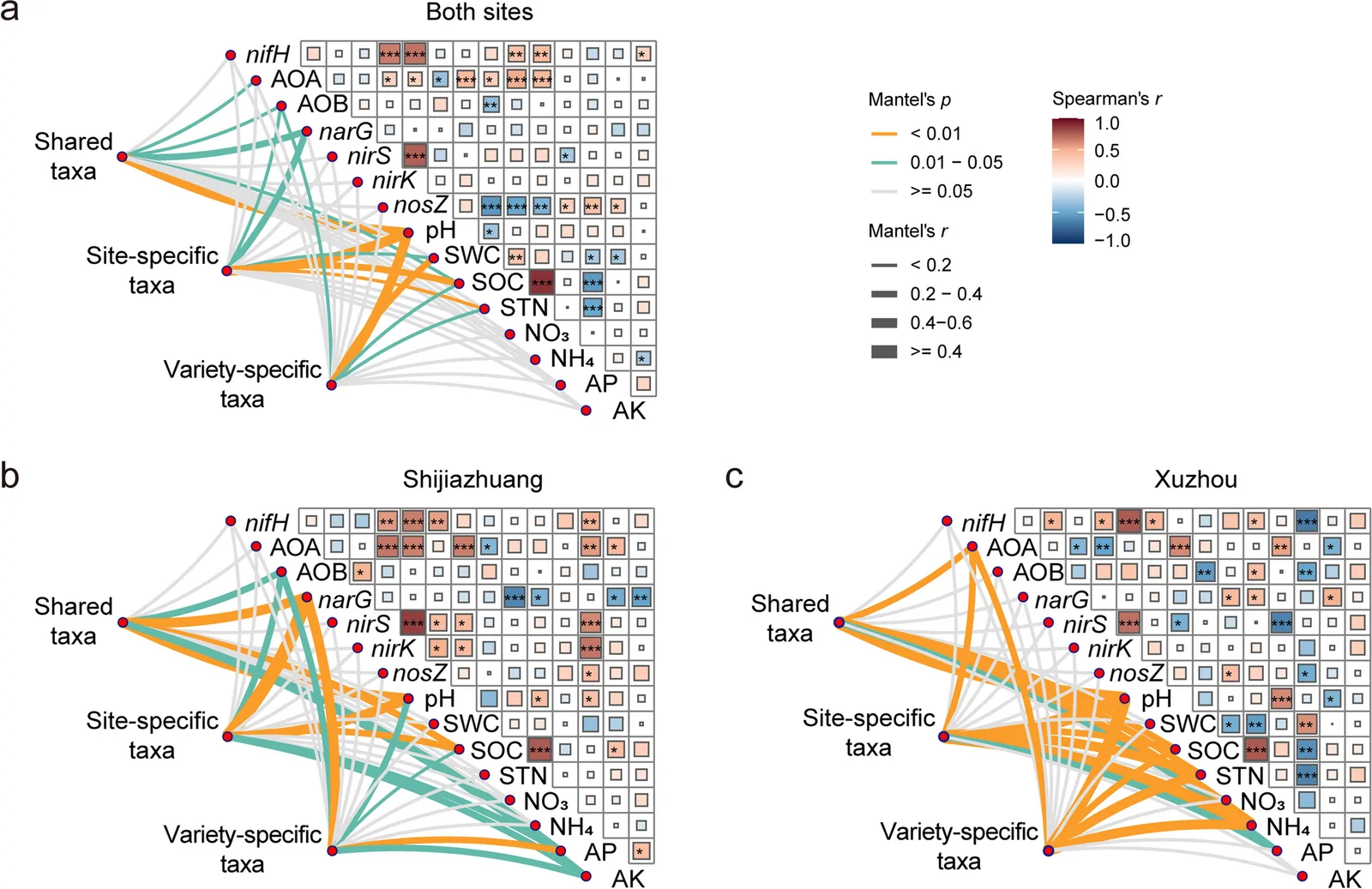
Relationships of the shared, site-specific, and variety-specific taxa with soil properties and N-cycle-related genes at **a** both sites, **b** Shijiazhuang, and **c** Xuzhou. *, **, and ** indicate significant differences at *p* < 0.05, 0.01, and 0.001, respectively. AOA archaeal *amoA*, AOB bacterial *amoA*, SWC soil water content, SOC soil organic carbon, STN soil total nitrogen, AP available phosphorus, AK available potassium

To further explore the interplay among soil properties, shared and site- and variety-specific taxa, and community N-cycle functions, their relationships were examined (Fig. 6). When both sites were included, shared taxa were correlated with archaeal and bacterial *amoA* and *narG*, site-specific taxa were correlated with bacterial *amoA* and *narG*, and variety-specific taxa were correlated with bacterial *amoA*. Shared and site- and variety-specific taxa were all correlated with pH and soil organic carbon, while only site-specific and variety-specific taxa were correlated with soil water content and total N. When the two sites were analyzed separately, the shared and site- and variety-specific taxa did not show much difference between each other except for the correlation with available P. They correlated with bacterial *amoA* and *narG* at Shijiazhuang and with archaeal *amoA* at Xuzhou, with pH and soil organic carbon at both sites, with available K at Shijiazhuang, and with soil total N and NH_4_ at Xuzhou. Available P was correlated with variety-specific taxa at Shijiazhuang and shared and site-specific taxa at Xuzhou.

## Discussion

### Soybean rhizosphere effect on the composition and N-cycling function of the bacterial community

The rhizosphere effect was found to be a major determinant in modulating the bacterial community in this study. The soybean rhizosphere had a substantially reduced PD whole tree index, and 1097–1471 OTUs were significantly depleted while only 443–613 were enriched (Figs. 1b and 2a). These results confirmed the strong selective role of the crop rhizosphere, which leads to a less diverse but more specialized community (Berendsen et al. 2012; Ling et al. 2022). Among the orders enriched in all soybean rhizosphere at both sites, *Rhizobiales* and *Bacillales* possess potential functions in N cycling, growth promotion, and disease suppression (Han et al. 2020; Xing et al. 2022). Furthermore, the small but significant effects of experimental sites and soybean varieties (Fig. 1b) together with the site-specific and variety-specific enriched orders (Supplemental Table S3) suggested a complex interaction between soybean root exudate profiles and the local microbiome (Zhalnina et al. 2018).

The differential composition of diazotrophs and abundances of N-cycle-related genes revealed that the soybean rhizosphere effect significantly modulates the functional potential of N cycling (Figs. 4a and b and 5a and Supplemental Fig. S1). The significant enrichment of N-cycling genes such as *nifH*, archaeal *amoA*, and *nosZ* indicated higher potential activity of nitrogen fixation, nitrification, and denitrification, suggesting that soybean rhizosphere created a microenvironment that promotes higher potential activity for N-cycling functions. These results are consistent with the metagenomics results reported by Mendes et al. (2014), which revealed a high abundance of genes associated with N fixation, denitrification, and nitrate/nitrite ammonification in the soybean rhizosphere. However, they partially deviate from the generalized rhizosphere effect on N-cycling functions demonstrated by a synthesis analysis of published sequences worldwide, which includes the enrichment of N fixation and denitrification microbes and the strong depletion of nitrification microbes (Ling et al. 2022). This inconsistency may be attributable to the influence of local soil conditions such as soil N availability, water content, and texture (Séneca et al. 2020; Lian et al. 2023). The influence of these local soil conditions could impact the rhizosphere’s ability to shape N-cycling microbial consortia (Ma et al. 2023), which is also revealed by the profound effects of the experimental site (explaining 47% of the total variation) on the diazotroph community (Fig. 4b) and the variable rhizosphere effects on the abundances of bacterial *amoA*, *narG*, *nirS*, and *nirK* (Fig. 5a). Furthermore, the rhizospheres of soybean varieties exhibited variations in the enrichment of the diazotroph orders *Rhizobiales* and *Pseudomonadales* (Fig. 4a) and the N-cycling-related functions nitrate/nitrite ammonification, N/nitrate respiration, and denitrification (Supplemental Fig. S1). These results suggested that the soybean genotype affected the recruitment of microbes with specific N-cycle-related functions in the soybean rhizosphere. This might be attributed to the differences in the growth and nutrient requirements of these soybean varieties, which may result in the differences in their root exudates. However, the regulatory mechanisms by which soybean genotypes control the recruitment of N-cycle-related microbes in the rhizosphere remain to be elucidated, potentially through detailed profiling of the root exudates of different soybean varieties.

### The taxonomy of the shared and site- and variety-specific taxa enriched in the soybean rhizosphere

Among the soybean rhizosphere-enriched OTUs, the shared taxa across all samples and those that were specific to sites and soybean varieties were identified, and they exhibited distinct compositions and correlated differently to soil properties (Figs. 2, 3, and 6).

In the shared taxa, most OTUs belonged to *Actinobacteria*, and the most abundance belonged to *Thaumarchaeota* (Fig. 3a). *Actinobacteria* are adept at decomposing complex organic compounds (Goodfellow and Williams 1983), which has also been previously reported to be enriched in the soybean rhizosphere (Mendes et al. 2014). *Thaumarchaeota* includes numerous ammonia-oxidizing archaea (Leininger et al. 2006) and contributes to maintaining microbial stability under continuous cropping of soybeans (Liu et al. 2022). The enrichment of shared taxa was correlated with the higher pH and soil organic carbon in the rhizosphere (Fig. 6a), which was in line with previous studies highlighting the vital role of soil pH and the organic carbon provided by soybean roots in attracting specific bacterial taxa (Bulgarelli et al. 2013).

Site-specific taxa exhibited significant variations between Shijiazhuang and Xuzhou, and they were dominated by *Actinobacteria* in Shijiazhuang but by *Nitrospirae* in Xuzhou (Fig. 3b, d). This differentiation could be linked to the different alterations of soil properties in the soybean rhizosphere between the two sites (Table 1). In Shijiazhuang, soybean rhizosphere exhibited an increase in the concentration of available phosphorus, which contrasted with a decrease in Xuzhou. Meanwhile, the rhizosphere showed a reduction in ammonium content in Xuzhou, which was not observed in Shijiazhuang. Notably, both available phosphorus and ammonium contents were found to correlate significantly with the respective site-specific taxa at each site (Fig. 6b, c).* Actinobacteria* is known as a phylum that includes many taxa with phosphorus-solubilizing capabilities (Mitra et al. 2022), which could account for the elevated available phosphorus levels in soybean rhizosphere in Shijiazhuang. Meanwhile, *Nitrospirae* comprises nitrifying bacteria, and some strains within this phylum have even been reported to be capable of completing the entire nitrification process independently, converting ammonium to nitrate (Daims et al. 2015), which might explain the reduction of ammonium content in the rhizosphere in Xuzhou. Therefore, the differences in these soil properties may not be causal factors but rather the consequences of the distinct site-specific taxa of each site.

Variety-specific taxa exhibited a more even distribution across several phyla (Fig. 3c, e), revealing the specific preferences of different soybean varieties when selecting microbes. Interestingly, a large proportion of these taxa had low relative abundances, with 391 out of 480 OTUs in Shijiazhuang and 358 out of 460 OTUs in Xuzhou presenting a relative abundance of less than 0.1‰ (Supplemental Table S5). As reported by Chen et al. (2020), rare microbial taxa were the major drivers of soil multifunctionality, and these low-abundance taxa may play specialized but potentially crucial roles in N cycling. Besides the soil properties that were also correlated with the shared and site-specific taxa, the variety-specific taxa were uniquely correlated with available phosphorus in Shijiazhuang. As phosphorus is known as an immobile and limiting nutrient that can influence N fixation and other N-cycling processes (Tang et al. 2020), this correlation indicated that the variety-specific taxa might contribute to the N-cycling functions by enhancing the availability of necessary nutrients such as phosphorus.

### The relationship between N-cycle-related functions and the shared, site-specific, and variety-specific taxa enriched in the soybean rhizosphere

The shared and site- and variety-specific taxa were closely related to the potential N-cycling function of the bacterial community in the soybean rhizosphere, as indicated by the network, random forest, and Mantel test in this study (Figs. 4c, 5c, and 6). This is consistent with previous studies reporting that rhizosphere-enriched taxa are intricately linked to several processes that are pivotal for crop growth, including the N cycle (Schmidt et al. 2019; Wei et al. 2015). However, taking the shared and site- and variety-specific taxa enriched in the soybean rhizosphere together, only 19 out of 1090 OTUs were annotated with N-cycle-related functions (Fig. 5b). This suggested that the rest of the OTUs in shared, site-, and variety-specific taxa might contribute to the N cycle in the soybean rhizosphere through interacting with these functional microbes even though they did not participate in N transformations themselves.

In the network with diazotrophs, positive correlations of the shared and site- and variety-specific taxa were found with only eight out of 39 diazotroph orders, including *Rhizobiales* (Fig. 4c). More negative correlations than positive correlations indicated complex competition between diazotrophs and other bacterial taxa (Faust and Raes 2012), which may help soybean plants recruit a microbiome that can enhance N fixation while limiting energy costs to support their nutrient needs. These results suggest that shared and site- and variety-specific taxa may contribute to this process by competing with diazotrophs that are less beneficial to soybean growth (Chepsergon and Moleleki 2023; Wheatley et al. 2020), potentially improving the efficiency of N fixation and reducing the need for chemical fertilizers. Furthermore, the variety-specific taxa exhibited a higher average degree than shared and site-specific taxa, indicating a strong, fine-tuned interaction with specific soybean genotypes, potentially facilitating optimized N fixation and utilization that is unique to each variety. Nevertheless, direct evidence for the improved N fixation of soybean varieties due to rhizosphere-enriched taxa is still needed.

The shared, site-specific, and variety-specific taxa did not show substantial differences in their impacts on the abundances of N-cycle-related genes (Fig. 5c). In contrast, their association with N-cycle-related genes was distinct between Shijiazhuang and Xuzhou, where they were negatively correlated with bacterial *amoA* and *narG* in Shijiazhuang but positively correlated with archaeal *amoA* in Xuzhou (Figs. 5c and 6). Despite the shared taxa being consistently enriched in the soybean rhizosphere across both sites, the ratio of ammonium (the substrate for ammonia-oxidation) to nitrate (the substrate for denitrification) was much lower in Shijiazhuang than in Xuzhou. This site-specific correlation indicated that they interacted with ammonia-oxidizing and denitrifying microbes differentially according to the relative levels of ammonium and nitrate. This result suggested their potential role in modulating N-cycle processes, which aligned with previous studies highlighting the role of the core microbiota in sustaining the stability of rhizosphere functionality (Jiao et al. 2022a, b). Site-specific taxa showed similar correlation patterns with N-cycle-related genes as shared taxa, suggesting that these taxa may act synergistically or complement the functions of the shared taxa, contributing to the stability and efficiency of N cycling within the rhizosphere (Saleem et al. 2019). A greater number of OTUs in variety-specific taxa were found in the most influential OTUs that affected the community N-cycling function in the random forest model at both sites, indicating that variety-specific taxa may play a relatively more important role in enhancing the N cycle. This underscored the influence of crop genotype on N-cycle processes (Favela et al. 2021), which may recruit unique taxa with redundant functions with those of the shared taxa, ensuring the robustness of important functions such as those involved in the N cycle. These results revealed the complex interplay between the shared, site-specific, and variety-specific taxa and N cycling. While the shared taxa provide a foundational contribution to maintaining N-cycle functionality, site-specific and variety-specific taxa provide supplementary resilience and adaptability to environmental variability.

Overall, this study of the bacterial community and N-cycling function in the rhizosphere of five typical soybean varieties grown in Shijiazhuang and Xuzhou demonstrated distinct compositions but similar associations with the potential N-cycling function of the shared, site-specific, and variety-specific taxa recruited by soybean roots. The results advance our understanding of the resilience and adaptability of the rhizosphere microbiota and its functions and provide potential insights for developing strategies to harness the rhizosphere microbiome for sustainable agriculture. The underlying mechanisms of the complex interactions between rhizosphere-enriched taxa and the N-cycle-related microbes need further investigation.

## Supplementary Information

Below is the link to the electronic supplementary material.

Supplementary file1 (PDF 657 KB)

Supplementary file2 (XLSX 148 KB)

## Data Availability

The raw sequences of the amplicon sequencing of 16S rRNA and *nifH* genes were deposited in the NCBI repository under BioProject accession number PRJNA1045824. The datasets generated during the analysis in the current study are available from the corresponding author on reasonable request.

## References

[CR1] Andrews S (2014) FastQC a quality control tool for high throughput sequence data. https://bioinformatics.babraham.ac.uk/projects/fastqc/

[CR2] Berendsen RL, Pieterse CMJ, Bakker PAHM (2012) The rhizosphere microbiome and plant health. Trends Plant Sci 17:478–486. 10.1016/j.tplants.2012.04.001 22564542 10.1016/j.tplants.2012.04.001

[CR3] Berg G, Smalla K (2009) Plant species and soil type cooperatively shape the structure and function of microbial communities in the rhizosphere. FEMS Microbiol Ecol 68:1–13. 10.1111/j.1574-6941.2009.00654.x 19243436 10.1111/j.1574-6941.2009.00654.x

[CR4] Bulgarelli D, Schlaeppi K, Spaepen S, Loren V, van Themaat E, Schulze-Lefert P (2013) Structure and functions of the bacterial microbiota of plants. Annu Rev Plant Biol 64:807–838. 10.1146/annurev-arplant-050312-120106 23373698 10.1146/annurev-arplant-050312-120106

[CR5] Bulgarelli D, Garrido-Oter R, Munch PC, Weiman A, Droge J, Pan Y, McHardy AC, Schulze-Lefert P (2015) Structure and function of the bacterial root microbiota in wild and domesticated barley. Cell Host Microbe 17:392–403. 10.1016/j.chom.2015.01.011 25732064 10.1016/j.chom.2015.01.011 PMC4362959

[CR6] Castellano-Hinojosa A, Strauss SL (2021) Insights into the taxonomic and functional characterization of agricultural crop core rhizobiomes and their potential microbial drivers. Sci Rep 11:10068. 10.1038/s41598-021-89569-7 33980901 10.1038/s41598-021-89569-7 PMC8115259

[CR7] Chang J, Tian L, Leite MFA, Sun Y, Shi S, Xu S, Wang J, Chen H, Chen D, Zhang J, Tian C, Kuramae EE (2022) Nitrogen, manganese, iron, and carbon resource acquisition are potential functions of the wild rice *Oryza rufipogon* core rhizomicrobiome. Microbiome 10:196. 10.1186/s40168-022-01360-6 36419170 10.1186/s40168-022-01360-6 PMC9682824

[CR8] Chen QL, Ding J, Zhu D, Hu HW, Delgado-Baquerizo M, Ma YB, He JZ, Zhu YG (2020) Rare microbial taxa as the major drivers of ecosystem multifunctionality in long-term fertilized soils. Soil Biol Biochem 141:107686. 10.1016/j.soilbio.2019.107686

[CR9] Chepsergon J, Moleleki LN (2023) Rhizosphere bacterial interactions and impact on plant health. Curr Opin Microbiol 73:102297. 10.1016/j.mib.2023.102297 37002974 10.1016/j.mib.2023.102297

[CR10] Daims H, Lebedeva EV, Pjevac P, Han P, Herbold C, Albertsen M, Jehmlich N, Palatinszky M, Vierheilig J, Bulaev A, Kirkegaard RH, von Bergen M, Rattei T, Bendinger B, Nielsen PH, Wagner M (2015) Complete nitrification by *Nitrospira* bacteria. Nature 528:504–509. 10.1038/nature16461 26610024 10.1038/nature16461 PMC5152751

[CR11] Edgar RC (2010) Search and clustering orders of magnitude faster than BLAST. Bioinformatics 26:2460–2461. 10.1093/bioinformatics/btq461 20709691 10.1093/bioinformatics/btq461

[CR12] Edwards J, Johnson C, Santos-Medellin C, Lurie E, Podishetty NK, Bhatnagar S, Eisen JA, Sundaresan V (2015) Structure, variation, and assembly of the root-associated microbiomes of rice. Proc Natl Acad Sci U S A 112:E911-920. 10.1073/pnas.1414592112 25605935 10.1073/pnas.1414592112 PMC4345613

[CR13] Faust K, Raes J (2012) Microbial interactions: from networks to models. Nat Rev Microbiol 10:538–550. 10.1038/nrmicro2832 22796884 10.1038/nrmicro2832

[CR14] Favela A, Bohn MO, Kent AD (2021) Maize germplasm chronosequence shows crop breeding history impacts recruitment of the rhizosphere microbiome. ISME J 15:2454–2464. 10.1038/s41396-021-00923-z 33692487 10.1038/s41396-021-00923-z PMC8319409

[CR15] Goodfellow M, Williams ST (1983) Ecology of actinomycetes. Annu Rev Microbiol 37:189–216. 10.1146/annurev.mi.37.100183.001201 6357051 10.1146/annurev.mi.37.100183.001201

[CR16] Green MR, Sambrook J (2019) Screening bacterial colonies using X-Gal and IPTG: α-complementation. Cold Spring Harb Protoc 2019. 10.1101/pdb.prot101329 10.1101/pdb.prot101329 31792144

[CR17] Han Q, Ma Q, Chen Y, Tian B, Xu L, Bai Y, Chen W, Li X (2020) Variation in rhizosphere microbial communities and its association with the symbiotic efficiency of rhizobia in soybean. ISME J 14:1915–1928. 10.1038/s41396-020-0648-9 32336748 10.1038/s41396-020-0648-9 PMC7367843

[CR18] Jiao S, Chen W, Wei G (2022a) Core microbiota drive functional stability of soil microbiome in reforestation ecosystems. Glob Change Biol 28:1038–1047. 10.1111/gcb.16024 10.1111/gcb.16024 34862696

[CR19] Jiao S, Qi J, Jin C, Liu Y, Wang Y, Pan H, Chen S, Liang C, Peng Z, Chen B, Qian X, Wei G (2022b) Core phylotypes enhance the resistance of soil microbiome to environmental changes to maintain multifunctionality in agricultural ecosystems. Glob Change Biol 28:6653–6664. 10.1111/gcb.16387 10.1111/gcb.16387 36002985

[CR20] Jin T, Wang Y, Huang Y, Xu J, Zhang P, Wang N, Liu X, Chu H, Liu G, Jiang H, Li Y, Xu J, Kristiansen K, Xiao L, Zhang Y, Zhang G, Du G, Zhang H, Zou H, Zhang H, Jie Z, Liang S, Jia H, Wan J, Lin D, Li J, Fan G, Yang H, Wang J, Bai Y, Xu X (2017) Taxonomic structure and functional association of foxtail millet root microbiome. Gigascience 6:1–12. 10.1093/gigascience/gix089 29050374 10.1093/gigascience/gix089 PMC7059795

[CR21] Kelly C, Haddix ML, Byrne PF, Cotrufo MF, Schipanski ME, Kallenbach CM, Wallenstein MD, Fonte SJ (2022) Long-term compost amendment modulates wheat genotype differences in belowground carbon allocation, microbial rhizosphere recruitment and nitrogen acquisition. Soil Biol Biochem 172:108768. 10.1016/j.soilbio.2022.108768

[CR22] Leininger S, Urich T, Schloter M, Schwark L, Qi J, Nicol GW, Prosser JI, Schuster SC, Schleper C (2006) Archaea predominate among ammonia-oxidizing prokaryotes in soils. Nature 442:806–809. 10.1038/nature04983 16915287 10.1038/nature04983

[CR23] Letunic I, Bork P (2021) Interactive Tree Of Life (iTOL) v5: an online tool for phylogenetic tree display and annotation. Nucleic Acids Res 49:W293–W296. 10.1093/nar/gkab301 33885785 10.1093/nar/gkab301 PMC8265157

[CR24] Lian T, Cheng L, Liu Q, Yu T, Cai Z, Nian H, Hartmann M (2023) Potential relevance between soybean nitrogen uptake and rhizosphere prokaryotic communities under waterlogging stress. ISME Commun 3:71. 10.1038/s43705-023-00282-0 37433864 10.1038/s43705-023-00282-0 PMC10336055

[CR25] Ling N, Wang T, Kuzyakov Y (2022) Rhizosphere bacteriome structure and functions. Nat Commun 13:836. 10.1038/s41467-022-28448-9 35149704 10.1038/s41467-022-28448-9 PMC8837802

[CR26] Liu Z, Liu J, Yu Z, Li Y, Hu X, Gu H, Li L, Jin J, Liu X, Wang G (2022) Archaeal communities perform an important role in maintaining microbial stability under long term continuous cropping systems. Sci Total Environ 838:156413. 10.1016/j.scitotenv.2022.156413 35660449 10.1016/j.scitotenv.2022.156413

[CR27] Louca S, Parfrey LW, Doebeli M (2016) Decoupling function and taxonomy in the global ocean microbiome. Science 353:1272–1277. 10.1126/science.aaf4507 27634532 10.1126/science.aaf4507

[CR28] Ma Y, Yue K, Heděnec P, Li C, Li Y, Wu Q (2023) Global patterns of rhizosphere effects on soil carbon and nitrogen biogeochemical processes. CATENA 220:106661. 10.1016/j.catena.2022.106661

[CR29] Mendes LW, Kuramae EE, Navarrete AA, van Veen JA, Tsai SM (2014) Taxonomical and functional microbial community selection in soybean rhizosphere. ISME J 8:1577–1587. 10.1038/ismej.2014.17 24553468 10.1038/ismej.2014.17 PMC4817605

[CR30] Mendes LW, Raaijmakers JM, de Hollander M, Mendes R, Tsai SM (2018) Influence of resistance breeding in common bean on rhizosphere microbiome composition and function. ISME J 12:212–224. 10.1038/ismej.2017.158 29028000 10.1038/ismej.2017.158 PMC5739014

[CR31] Mitra D et al (2022) Actinobacteria-enhanced plant growth, nutrient acquisition, and crop protection: advances in soil, plant, and microbial multifactorial interactions. Pedosphere 32:149–170. 10.1016/S1002-0160(21)60042-5

[CR32] Quast C, Pruesse E, Yilmaz P, Gerken J, Schweer T, Yarza P, Peplies J, Glöckner FO (2012) The SILVA ribosomal RNA gene database project: improved data processing and web-based tools. Nucleic Acids Res 41:D590–D596. 10.1093/nar/gks1219 23193283 10.1093/nar/gks1219 PMC3531112

[CR33] Reinhold-Hurek B, Bunger W, Burbano CS, Sabale M, Hurek T (2015) Roots shaping their microbiome: global hotspots for microbial activity. Annu Rev Phytopathol 53:403–424. 10.1146/annurev-phyto-082712-102342 26243728 10.1146/annurev-phyto-082712-102342

[CR34] Ren Y, Su L, Hou X, Shao J, Liu K, Shen Q, Zhang R, Xun W (2023) Rhizospheric compensation of nutrient cycling functions dominates crop productivity and nutrient use efficiency. Appl Soil Ecol 182:104722. 10.1016/j.apsoil.2022.104722

[CR35] Saleem M, Hu J, Jousset A (2019) More than the sum of its parts: microbiome biodiversity as a driver of plant growth and soil health. Annu Rev Ecol Evol Syst 50:145–168. 10.1146/annurev-ecolsys-110617-062605

[CR36] Sánchez C, Minamisawa K (2019) Nitrogen cycling in soybean rhizosphere: sources and sinks of nitrous oxide (N_2_O). Front Microbiol 10:1943. 10.3389/fmicb.2019.01943 31497007 10.3389/fmicb.2019.01943 PMC6712156

[CR37] Schmidt JE, Kent AD, Brisson VL, Gaudin ACM (2019) Agricultural management and plant selection interactively affect rhizosphere microbial community structure and nitrogen cycling. Microbiome 7:146. 10.1186/s40168-019-0756-9 31699148 10.1186/s40168-019-0756-9 PMC6839119

[CR38] Schultze M, Kondorosi A (1998) Regulation of symbiotic root nodule development. Annu Rev Genet 32:33–57. 10.1146/annurev.genet.32.1.33 9928474 10.1146/annurev.genet.32.1.33

[CR39] Séneca J, Pjevac P, Canarini A, Herbold CW, Zioutis C, Dietrich M, Simon E, Prommer J, Bahn M, Pötsch EM, Wagner M, Wanek W, Richter A (2020) Composition and activity of nitrifier communities in soil are unresponsive to elevated temperature and CO_2_, but strongly affected by drought. ISME J 14:3038–3053. 10.1038/s41396-020-00735-7 32770119 10.1038/s41396-020-00735-7 PMC7784676

[CR40] Tang W, Cerdán-García E, Berthelot H, Polyviou D, Wang S, Baylay A, Whitby H, Planquette H, Mowlem M, Robidart J, Cassar N (2020) New insights into the distributions of nitrogen fixation and diazotrophs revealed by high-resolution sensing and sampling methods. ISME J 14:2514–2526. 10.1038/s41396-020-0703-6 32581316 10.1038/s41396-020-0703-6 PMC7490393

[CR41] Thiergart T, Durán P, Ellis T, Vannier N, Garrido-Oter R, Kemen E, Roux F, Alonso-Blanco C, Ågren J, Schulze-Lefert P, Hacquard S (2020) Root microbiota assembly and adaptive differentiation among European *Arabidopsis* populations. Nat Ecol Evol 4:122–131. 10.1038/s41559-019-1063-3 31900452 10.1038/s41559-019-1063-3

[CR42] Trivedi P, Leach JE, Tringe SG, Sa T, Singh BK (2020) Plant–microbiome interactions: from community assembly to plant health. Nat Rev Microbiol 18:607–621. 10.1038/s41579-020-0412-1 32788714 10.1038/s41579-020-0412-1

[CR43] Tsiknia M, Tsikou D, Papadopoulou KK, Ehaliotis C (2020) Multi-species relationships in legume roots: From pairwise legume-symbiont interactions to the plant – microbiome – soil continuum. FEMS Microbiol Ecol 97. 10.1093/femsec/fiaa222 10.1093/femsec/fiaa222 33155054

[CR44] Walters WA, Jin Z, Youngblut N, Wallace JG, Sutter J, Zhang W, Gonzalez-Pena A, Peiffer J, Koren O, Shi Q, Knight R, Glavina Del Rio T, Tringe SG, Buckler ES, Dangl JL, Ley RE (2018) Large-scale replicated field study of maize rhizosphere identifies heritable microbes. Proc Natl Acad Sci U S A 115:7368–7373. 10.1073/pnas.1800918115 29941552 10.1073/pnas.1800918115 PMC6048482

[CR45] Wei Z, Yang T, Friman V-P, Xu Y, Shen Q, Jousset A (2015) Trophic network architecture of root-associated bacterial communities determines pathogen invasion and plant health. Nat Commun 6:8413. 10.1038/ncomms9413 26400552 10.1038/ncomms9413 PMC4598729

[CR46] Wen T, Xie P, Yang S, Niu G, Liu X, Ding Z, Xue C, Liu Y-X, Shen Q, Yuan J (2022) ggClusterNet: An R package for microbiome network analysis and modularity-based multiple network layouts. iMeta 1:e32. 10.1002/imt2.32 38868720 10.1002/imt2.32 PMC10989811

[CR47] Wheatley RM, Ford BL, Li L, Aroney STN, Knights HE, Ledermann R, East AK, Ramachandran VK, Poole PS (2020) Lifestyle adaptations of *Rhizobium* from rhizosphere to symbiosis. Proc Natl Acad Sci U S A 117:23823–23834. 10.1073/pnas.2009094117 32900931 10.1073/pnas.2009094117 PMC7519234

[CR48] Xing PF, Zhao YB, Guan DW, Li L, Zhao BS, Ma MC, Jiang X, Tian CF, Cao FM, Li J (2022) Effects of *Bradyrhizobium* co-inoculated with *Bacillus* and *Paenibacillus* on the structure and functional genes of soybean *Rhizobacteria* community. Genes 13. 10.3390/genes13111922 10.3390/genes13111922 PMC9689485 36360159

[CR49] Xu Y, Wang G, Jian J, Liu J, Zhang Q, Liu X (2009) Bacterial communities in soybean rhizosphere in response to soil type, soybean genotype, and their growth stage. Soil Biol Biochem 41:919–925. 10.1016/j.soilbio.2008.10.027

[CR50] Xu Y, Ge Y, Song JX, Rensing C (2020) Assembly of root-associated microbial community of typical rice cultivars in different soil types. Biol Fertil Soils 56:249–260. 10.1007/s00374-019-01406-2

[CR51] Yeoh YK, Dennis PG, Paungfoo-Lonhienne C, Weber L, Brackin R, Ragan MA, Schmidt S, Hugenholtz P (2017) Evolutionary conservation of a core root microbiome across plant phyla along a tropical soil chronosequence. Nat Commun 8:215. 10.1038/s41467-017-00262-8 28790312 10.1038/s41467-017-00262-8 PMC5548757

[CR52] Zhalnina K, Louie KB, Hao Z, Mansoori N, da Rocha UN, Shi S, Cho H, Karaoz U, Loque D, Bowen BP, Firestone MK, Northen TR, Brodie EL (2018) Dynamic root exudate chemistry and microbial substrate preferences drive patterns in rhizosphere microbial community assembly. Nat Microbiol 3:470–480. 10.1038/s41564-018-0129-3 29556109 10.1038/s41564-018-0129-3

[CR53] Zhang Y, Xu J, Riera N, Jin T, Li J, Wang N (2017) Huanglongbing impairs the rhizosphere-to-rhizoplane enrichment process of the citrus root-associated microbiome. Microbiome 5:97. 10.1186/s40168-017-0304-4 28797279 10.1186/s40168-017-0304-4 PMC5553657

[CR54] Zhang BG, Zhang J, Liu Y, Guo YQ, Shi P, Wei GH (2018) Biogeography and ecological processes affecting root-associated bacterial communities in soybean fields across China. Sci Total Environ 627:20–27. 10.1016/j.scitotenv.2018.01.230 29426141 10.1016/j.scitotenv.2018.01.230

[CR55] Zhang J, Liu Y-X, Zhang N, Hu B, Jin T, Xu H, Qin Y, Yan P, Zhang X, Guo X, Hui J, Cao S, Wang X, Wang C, Wang H, Qu B, Fan G, Yuan L, Garrido-Oter R, Chu C, Bai Y (2019) *NRT1.1B* is associated with root microbiota composition and nitrogen use in field-grown rice. Nat Biotechnol. 10.1038/s41587-019-0104-4 10.1038/s41587-019-0104-4 31036930

[CR56] Zheng T, Li Y, Li Y, Zhang S, Ge T, Wang C, Zhang F, Faruquee M, Zhang L, Wu X, Tian Y, Jiang S, Xu J, Qiu L (2022) A general model for “germplasm-omics” data sharing and mining: a case study of SoyFGB v2.0. Sci Bull (beijing) 67:1716–1719. 10.1016/j.scib.2022.08.001 36546052 10.1016/j.scib.2022.08.001

