# Supplementary material for: Dissecting the role of soybean rhizosphere-enriched bacterial taxa in modulating nitrogen-cycling functions
Source: Appl Microbiol Biotechnol. 2024 May 28;108:347. doi: 10.1007/s00253-024-13184-5 (PMC11133221; doi:10.1007/s00253-024-13184-5)
Supplement: Supplementary file 1 [file 253_2024_13184_MOESM1_ESM.pdf]

Supplemental material

Journal: Applied Microbiology and Biotechnology

Dissecting the role of soybean rhizosphere-enriched bacterial taxa in modulating nitrogen-cycling functions

Tianshu Wang[1#] · Miao Gao[1#] · Weiwei Shao[1] · Li Wang[1] · Chunyan Yang[2] · Xing Wang[3] · Shuihong Yao[1] · Bin Zhang[4*] 1 State Key Laboratory of Efficient Utilization of Arid and Semi-arid Arable Land in Northern China (the Institute of Agricultural Resources and Regional Planning, Chinese Academy of Agricultural Sciences, Beijing 100081, China)*
2 The Key Laboratory of Crop Genetics and Breeding of Hebei, Institute of Cereal and Oil Crops, Hebei Academy of Agricultural and Forestry Sciences, Shijiazhuang 050031, China
3 Jiangsu Xuhuai Regional Institute of Agricultural Sciences, Xuzhou 221131, China
4 College of Resources and Environmental Sciences, Nanjing Agricultural University, Nanjing 210095, China

The authors contributed to the work equally.

*Corresponding author: Email: yaoshuihong@caas.cn; zhangbin10@njau.edu.cn

Table S1 The accession number, name, type, growth habit, and stem termination of the five varieties in this study (Zheng et al. 2022).

Accession number	Variety	Type	Growth habit	Stem termination
ZDD03460	Deng-feng-chang-yang-hei-dou (DF)	Wild	Semi-sprawl	Indeterminate
ZDD03538	Huo-jia-hong-hei-dou (HJ)	Admixture	Erect	Determinate
ZDD11395	Meng-cheng-bai-jian-ke (MC)	Admixture	Semi-sprawl	Indeterminate
ZDD11396	Chu-xian-da-bai-ke (CX)	Cultivated	Semi-erect	Semi-determinate
ZDD25129	Qu-xian-3-hao (QX)	Cultivated	Erect	Determinate

Table S2 The name, function, and qPCR primers of the genes quantified in this study.

Gene name	Function	Primers	Reference
nifH	Nitrogenase reductase	PolF: TGCGAYCCSAARGCBGACTC<br>PolR: ATSGCCATCATYTCRCCGGA	(Poly et al. 2001)
Archaeal *amoA*	Ammonia monooxygenase	Arch-amoAF: STAATGGTCTGGCTTAGACG<br>Arch-amoAR: GCGGCCATCCATCTGTATGT	(Francis et al. 2005)
Bacterial *amoA*	Ammonia monooxygenase	amoA-1F: GGGGTTTCTACTGGTGGT<br>amoA-1R: CCCCTCKGSAAAGCCTTCTTC	(Rotthauwe et al. 1997)
narG	Nitrate reductase	nirG-f: TCGCCSATYCCGGCSATGTC<br>nirG-R: GAGTTGTACCAGTCRGCSGAYTCSG	(Bru et al. 2007)
nirS	Nitrite reductase	Cd3aF: GTSAACGTSAAGGARACSGG<br>R3cd: GASTTCGGRTGSGTCTTGA	(Hallin and Lindgren 1999; Michotey et al. 2000)
nirK	Copper-containing nitrite reductase	F1aCu: ATCATGGTSCTGCCGCG<br>R3Cu: GCCTCGATCAGRTTGTGGTT	(Kandeler et al. 2006)
nosZ	Nitrous-oxide reductase	nosZ 2F: CGCRACGGCAASAAGGTSMSSGT<br>nosZ 2R: CAKRTGCAKSGCRTGGCAGAA	(Henry et al. 2006)

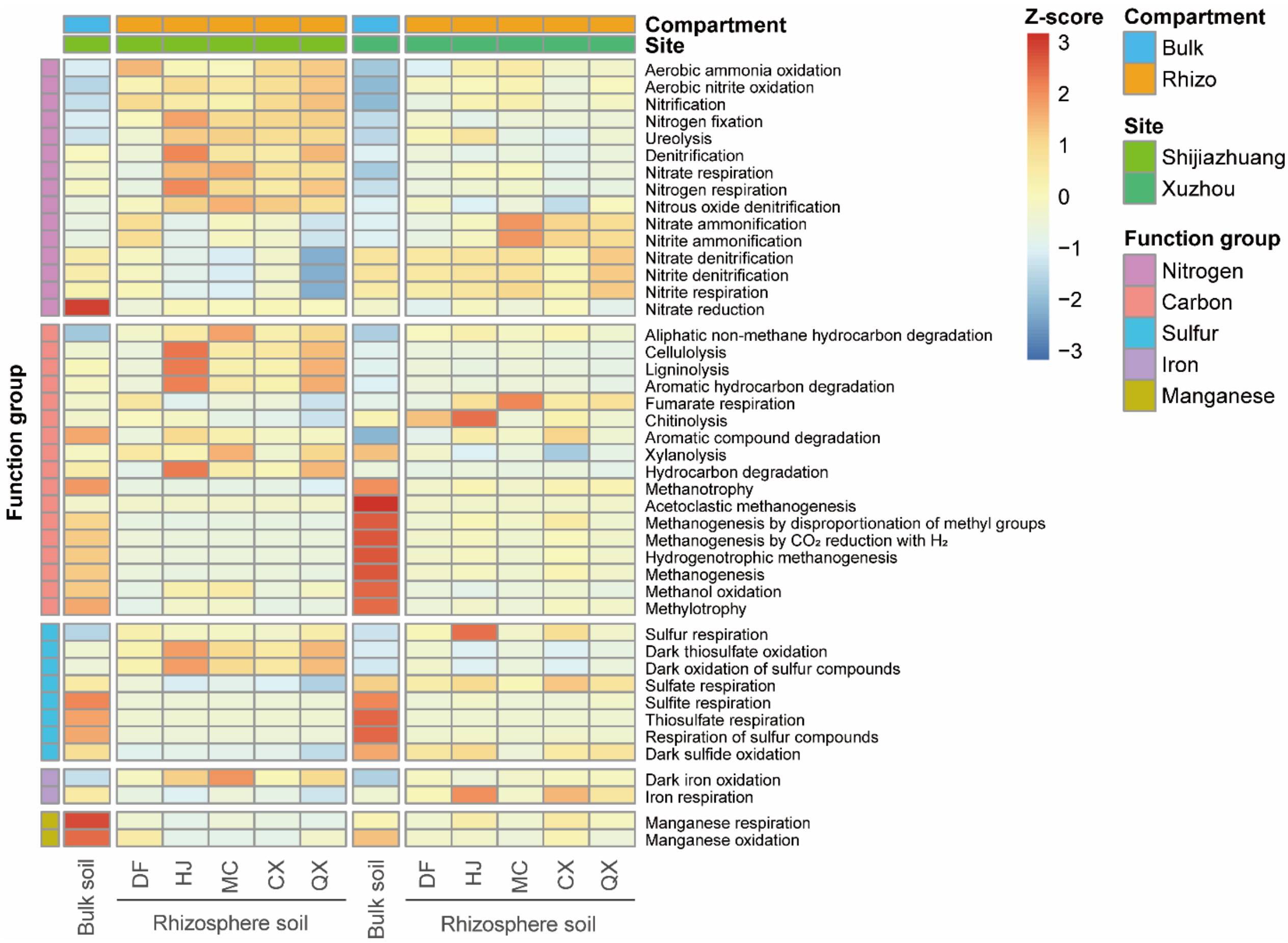

Fig. S1 The ecological function of the bacterial communities in bulk soil and the rhizosphere of five varieties in Shijiazhuang and Xuzhou as predicted by FAPROTAX.

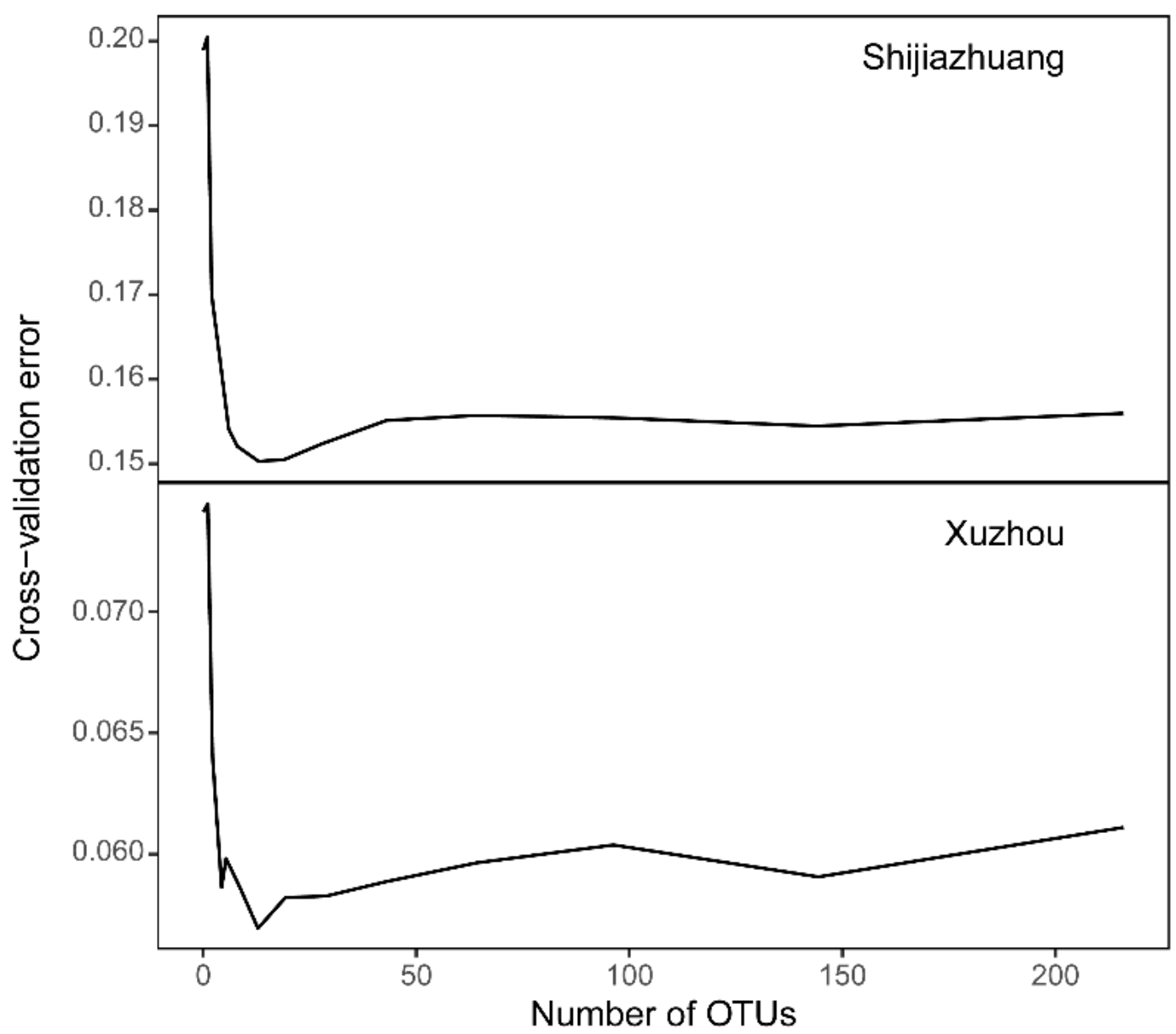

Fig. S2 The average ten-fold cross-validation error (N = 5) of the number of OTUs in the randomforest model in Shijiazhuang and Xuzhou.

Bru D, Sarr A, Philippot L (2007) Relative abundances of proteobacterial membrane-bound and periplasmic nitrate reductases in selected environments. Appl Environ Microbiol 73:5971-5974. https://doi.org/10.1128/aem.00643-07

Francis CA, Roberts KJ, Beman JM, Santoro AE, Oakley BB (2005) Ubiquity and diversity of ammonia-oxidizing archaea in water columns and sediments of the ocean. Proc Natl Acad Sci 102:14683-14688. https://doi.org/10.1073/pnas.0506625102

Hallin S, Lindgren PE (1999) PCR detection of genes encoding nitrite reductase in denitrifying bacteria. Appl Environ Microbiol 65:1652-1657. https://doi.org/10.1128/aem.65.4.1652-1657.1999

Henry S, Bru D, Stres B, Hallet S, Philippot L (2006) Quantitative detection of the *nosZ* gene, encoding nitrous oxide reductase, and comparison of the abundances of 16S rRNA, *narG*, *nirK*, and *nosZ* genes in soils. Appl Environ Microbiol 72:5181-5189. https://doi.org/10.1128/aem.00231-06

Kandeler E, Deiglmayr K, Tscherko D, Bru D, Philippot L (2006) Abundance of *narG*, *nirS*, *nirK*, and *nosZ* genes of denitrifying bacteria during primary successions of a glacier foreland. Appl Environ Microbiol 72:5957-5962. https://doi.org/10.1128/aem.00439-06

Michotey V, Méjean V, Bonin P (2000) Comparison of methods for quantification of cytochrome cd(1)-denitrifying bacteria in environmental marine samples. Appl Environ Microbiol 66:1564-1571. https://doi.org/10.1128/aem.66.4.1564-1571.2000

Poly F, Monrozier LJ, Bally R (2001) Improvement in the RFLP procedure for studying the diversity of *nifH* genes in communities of nitrogen fixers in soil. Res Microbiol 152:95-103. https://doi.org/10.1016/S0923-2508(00)01172-4

Rotthauwe JH, Witzel KP, Liesack W (1997) The ammonia monooxygenase structural gene *amoA* as a functional marker: molecular fine-scale analysis of natural ammonia-oxidizing populations. Appl Environ Microbiol 63:4704-4712. https://doi.org/10.1128/aem.63.12.4704-4712.1997

Zheng T, Li Y, Li Y, Zhang S, Ge T, Wang C, Zhang F, Faruquee M, Zhang L, Wu X, Tian Y, Jiang S, Xu J, Qiu L (2022) A general model for "germplasm-omics" data sharing and mining: a case study of SoyFGB v2.0. Sci Bull (Beijing) 67:1716-1719. https://doi.org/10.1016/j.scib.2022.08.001
